# Genetic Association of *CYP1B1 4326 C>G* Polymorphism with Disease-Free Survival in TNBC Patients Undergoing TAC Chemotherapy Regimen

**DOI:** 10.31557/APJCP.2021.22.4.1319

**Published:** 2021-04

**Authors:** Ahmad Aizat Abdul Aziz, Md Salzihan Md Salleh, Maya Mazuwin Yahya, Andee Dzulkarnaen Zakaria, Ravindran Ankathil

**Affiliations:** 1 *Human Genome Centre, School of Medical Sciences, Universiti Sains Malaysia, Health Campus, Kubang Kerian, Kelantan, Malaysia. *; 2 *Department of Pathology School of Medical Sciences, Universiti Sains Malaysia, Health Campus, Kubang Kerian, Kelantan, Malaysia. *; 3 *Department of Surgery, School of Medical Sciences, Universiti Sains Malaysia, Health Campus, Kubang Kerian, Kelantan, Malaysia. *

**Keywords:** TNBC, CYP1B1, single nucleotide polymorphism, disease free survival

## Abstract

**Background:**

Triple negative breast cancer (TNBC) which is treated with taxane, adriamycin and cyclophosphamide (TAC) chemotherapy regimen show variation in treatment response. *CYP1B1 4326 C>G* polymorphism has been implicated in contributing to the differences in treatment response in various types of cancers.

**Aim:**

The objective of the present study was to investigate whether this polymorphism modulate the risk of disease recurrence in TNBC patients undergoing TAC chemotherapy regimen.

**Methods:**

Blood samples of 76 immunohistochemistry confirmed TNBC patients were recruited. The genotyping of *CYP1B1 4326 C>G* polymorphism was carried out using PCR-RFLP technique. The genotype patterns were categorized into homozygous wildtype, heterozygous and homozygous variant. Kaplan-Meier analysis followed by Cox proportional hazard regression model were performed to evaluate the TNBC patients’ recurrence risk.

**Results:**

Out of 76 TNBC patients, 25 (33.0%) showed disease recurrence after one-year evaluation. Kaplan Meier analysis showed that TNBC patients who are carriers of *CYP1B1 4326 GG *variant genotypes (37.0%) had a significantly lower probability of disease-free rates as compared to TNBC patients who are carriers of *CYP1B1 4326 CC/CG* genotypes (71.0%). Univariate and multivariate Cox analysis demonstrated that TNBC patients who carried *CYP1B1 4326 GG* variant genotype had a significantly higher risk of recurrence with HR: 2.50 and HR: 4.18 respectively, even after adjustment as compared to TNBC patients who were carriers of *CYP1B1 4326 CC *and *CG *genotypes.

**Conclusion:**

Our results demonstrate the potential use of *CYP1B1 4325 GG* variant genotype as a candidate biomarker in predicting risk of recurrence in TNBC patients undergoing TAC chemotherapy regimen.

## Introduction

Triple negative breast cancer (TNBC), an aggressive breast cancer subtype, is characterized by lack of expression of estrogen (ER), progesterone (PR) and human epidermal growth factors 2 (HER2) receptors (Elias, 2010). TNBC is associated with higher cell proliferation, invasion and metastasis with high rate of recurrence within the first three years of diagnosis (Foulkes et al., 2010; Wang et al., 2018). Due to lack of clinical therapy and molecular targets, adjuvant chemotherapy is the only available systemic treatment for TNBC. TNBC is sensitive to chemotherapeutic agents such as taxanes and anthracyclines (Lehmann et al., 2011; Bilici et al., 2012). However, due to tumour heterogeneity, 20-40% of early stage TNBC patients who are treated with anthracyclines-taxane based chemotherapy develop metastatic disease and die of the disease (Nielsen et al., 2004; Haffty et al., 2006). Only, 20-34% of TNBC patients achieve pathological complete response (pCR) (Liedtke et al., 2008; Von Minckwitz et al., 2012) 

Since TNBC lacks specific targets and clinical therapies, the availability of biomarkers that reliably differentiate between chemotherapy sensitive and resistant patients are required to improve chemotherapy. Genetic variations such as single nucleotide polymorphisms (SNPs) have been widely studied as potential biomarkers in predicting disease recurrence and treatment outcomes. SNPs in genes encoding drug metabolizing enzyme such as cytochrome (P450) may activate (or inactivate) the gene function activity, that can lead to deregulation of the drug adsorption, distribution, metabolism and excretion (ADME) (Iskakova et al., 2016) and consequently lead to therapeutic failures and/or adverse drug reactions.

Cytochrome P450 family 1 subfamily B member 1 (CYP1B1) is a gene that plays a role in the biotransformation of xenobiotics, endogenous compounds as well as chemotherapeutic drugs including taxanes and anthracyclines (Chun and Kim, 2003; Martinez et al., 2008). *CYP1B1 4326 C>G* polymorphism which is the commonest SNP in *CYP1B1* located at coding region of *CYP1B1* mRNA, leads to amino acid change at codon 432 from leucine to valine (Rizzo et al., 2010). A few studies have demonstrated that this polymorphism contributes to the differences in disease recurrence and treatment response in various types of cancers (Gehrmann et al., 2008; Pastina et al., 2010; Dumont et al., 2015; Vasile et al., 2015). However, study on the impact of *CYP1B1 4326 C>G* polymorphism in modulating disease recurrence on TNBC patients is limited. Thus, the present study was designed to investigate the impact of *CYP1B1 4326 C>G* polymorphism in modulating disease recurrence and treatment outcome among TNBC patients undergoing taxane, adriamycin and cyclophosphamide (TAC) chemotherapy regimen. 

## Materials and Methods

*Study subjects*

The study was approved by the Human Research Ethics Committee of Universiti Sains Malaysia (USMKK/PPP/JEPeM[260.39210]) and Ministry of Health, Malaysia (NMRR-15-1200-25230) which complies with Declaration of Helsinki. Breast cancer patients who were histopathologically confirmed as negative ER, PR hormone receptors by immunohistochemistry (IHC) staining and no amplification of HER 2 by fluorescence *in situ* hybridization (FISH), who had undergone surgical resection and completed six cycles of chemotherapy with TAC regimen were included in the study. The study subjects were recruited from Oncology and Radiotherapy Clinic, Hospital Universiti Sains Malaysia while analysis was carried out at the Human Genome Centre, Universiti Sains Malaysia, Kubang Kerian, Kelantan. Clinicopathological data of the TNBC patients such as age at diagnosis, tumour histology subtype, tumour staging, tumour histograde, axillary lymph node metastasis status were traced from the patients’ medical folders and recorded.

*CYP1B1 4326 C>G SNP genotyping*

Three (3) ml of peripheral blood was collected from the study subjects in sterile EDTA-coated tubes. DNA was extracted using QIAamp DNA mini Kit (Qiagen, Hilden, Germany). *CYP1B1 4326 C>G* (rs1056836) genotyping was performed by using polymerase chain reaction-restriction fragment length polymorphism (PCR-RFLP) technique using PCR primers (Forward: 5’ CTATGTCCTGGCCTTCCTTTAT 3’ and Reverse: 5’ TCTCTGAGAGTGACATTGACTT 3’) sequences which generated a 435 bp product containing the polymorphic site. The PCR reactions were carried out in a 25 μl volume of 1X.

**Figure 1:**
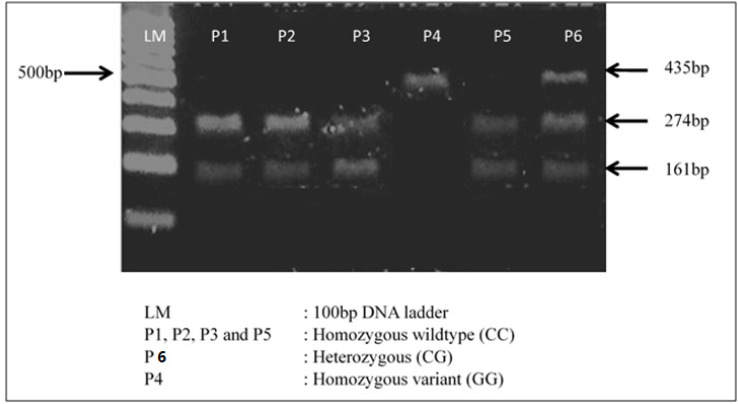
Gel Picture Showing Different Genotype Patterns of *CYP1B1 4326 C>G* Polymorphism in TNBC Patients

PCR Buffer, 2.0mM of MgCl_2_, 0.5mM dNTPs, 0.4mM of each primer, 1.25U of *GoTaq* polymerase and 50-100ng of DNA template with a denaturation of 95˚C for 2 min, followed by 35 cycles at 95˚C for 30 s, 55˚C for 30 s and 72˚C for 30 s and finally 5 min at 72 ˚C. Following amplification, PCR products were digested using *Alel *restriction enzyme for 1 hour at 37 ˚C and electrophoresed on 2% agarose gel. Samples which showed presence of two small fragments (274 and 161 bp) were identified as homozygous wildtype (CC) genotype. On the other hand, presence of one single band at 435 bp was identified as homozygous variant (GG) genotype, while presence of three bands at 435, 274 and 161 bp were identified as heterozygous variant (CG) genotype (Figure 1). 

*Evaluation of treatment response*

TNBC patients who had completed six cycles of chemotherapy with TAC regimen were evaluated after one year. Those TNBC patients who developed disease progression, local recurrence, primary and secondary tumour at different location were categorized under disease recurrence group, while those patients who did not show any signs above, were categorized under non-disease recurrence (chemoresponsive) group. The disease relapse was evaluated based on ultrasound, computed tomography (CT scan) or magnetic resonance imaging (MRI) findings by the treating oncologist. 

*Statistical analysis*

The cumulative disease-free survival (DFS) analysis of TNBC patients were performed and Kaplan-Meier method and the log-rank test were used for single-factor analysis. Univariate and multivariate analyses were performed using Cox proportional hazard regression model. In the present study, the DFS was calculated as the time from first chemotherapy to the first locoregional or distant recurrence as per Kim et al., (2015). 

## Results

The mean age of the 76 TNBC patients at diagnosis was 49.8 ± 9.87 years. The genotype frequencies of *CYP1B1 4326 C>G* polymorphism and clinicopathological features of TNBC patients are shown in Table 1. During follow up of the 76 TNBC patients, 25 (33.0%) presented disease recurrence and 51 (67.0%) showed no evidence of disease recurrence. Among the disease-recurrence group, 16 (64.0%) showed *CYP1B1 4326 CC/CG* genotypes and 9 (36.0%) showed *CYP1B1 4326 GG* genotype. Whereas, in non-disease recurrence group, 45 (88.0%) showed *CYP1B1 4326 CC/CG* genotypes and 6 (12.0%) showed *CYP1B1 4326 GG* genotype. The present study demonstrated that the *CYP1B1 4326 GG* homozygous variant genotype and positive axillary lymph nodes metastasis were significantly higher in disease recurrence as compared to non-disease recurrence group with p=0.006 and 0.027 respectively. Other clinicopathological variables did not show any significant differences between disease recurrence and non-disease recurrence groups. 

**Figure 2:**
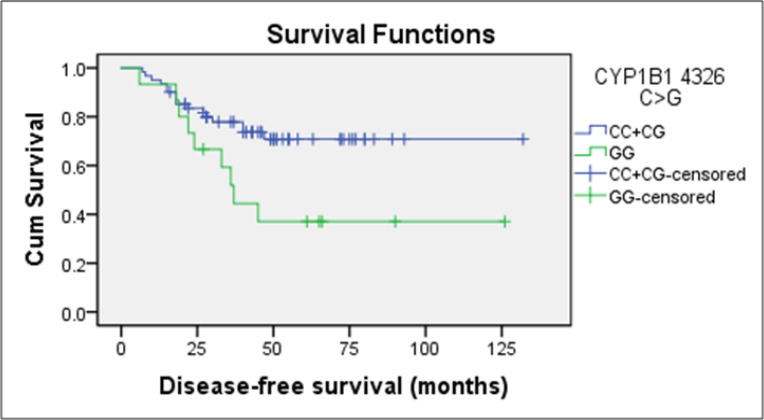
Kaplan Meier Analysis of *CYP1B1 4326 C>G* Polymorphism

Kaplan-Meier analysis showed that TNBC patients who are carriers of *CYP1B1 4326 GG* variant genotypes (37.0%) had a significantly lower probability of DFS as compared to TNBC patients who are carriers of *CYP1B1 4326 CC/CG* genotypes (71.0 %) respectively (p=0.023) (Figure 2). Further, univariate and multivariate Cox regression analysis showed that TNBC patients who are carriers of *CYP1B1 4326 GG* homozygous variant genotype had a significantly higher risk of relapse with HR= 2.50, (95% CI: 1.10,5.66) and HR= 4.18, (95% CI: 1.64,10.66), even after adjustment as compared to those TNBC patients who are carriers of *CYP1B1 4326 CC/CG* genotypes (Table 2). 

**Table 1:** *CYP1B1* 4326 C>G Polymorphism, Clinicopathological Characteristics and Association with Disease Recurrence

	Disease recurrence (N=25)	Non-disease recurrence (N=51)	p value
*CYP1B1 *4326 C>G			
CC/CG	16 (64.0%)	45 (88.0%)	
GG	9 (36.0%)	6 (12.0%)	0.006*
Histology subtype			
Infiltrating ductal carcinoma	17 (68.0%)	45 (88.0%)	
Other (medullary & metaplastic)	8 (32.0%)	6 (12.0%)	0.056
Tumor grading			
I and II	11 (44.0%)	22 (43.0%)	
III	14 (56.0%)	29 (57.0%)	1
Tumour staging			
I and II	18 (72.0%)	46 (90.0%)	
III	7 (28.0%)	5 (10.0%)	0.052
Lymph node metastasis			
Negative	7 (28.0%)	29 (57.0%)	
Positive	18 (72.0%)	22 (43.0%)	0.027*
Menopausal status			
Menopausal	7 (28.0%)	20 (39.0%)	
Pre-menopausal	18 (72.0%)	31 (61.0%)	0.446

*p-value < 0.05, statistically significant

**Table 2:** Univariate and Multivariate Cox Regression Analysis of TNBC Patients with Polymorphic Genotypes and Risk of Disease Recurrence

Polymorphism	Crude HR^a^	Adjusted HR^b^	Wald statistics (df)^b^	p value
*CYP1B1* 4326 C>G				
CC and CG	1 (Reference)	1 (Reference)		
GG	2.5 (1.10,5.66)	4.18 (1.64,10.66)	8.94	0.003*

^a^, Univariate Cox regression analysis; ^b^, Adjusted HR by histology subtype, grade, stage, axillary lymph node metastasis and menopausal status based on Multivariate Cox regression analysis; *p-value < 0.05, statistically significant

## Discussion

*CYP1B1* is an anticancer drug metabolizer and its polymorphism 4326 C>G has been reported to contribute to interindividual variation in drug efficacy and toxicity as well as treatment outcome. To the best of available knowledge, this is the first study investigating *CYP1B1 4326 C>G* polymorphism as a potential biomarker for predicting disease recurrence among Malaysian TNBC patients undergoing TAC chemotherapy regimen. In the present study, the mean age of TNBC patients at diagnosis ± (SD) was 48.9 ± 9.67 years. A recent study on Malaysian TNBC patients reported the range of age at diagnosis of Malaysian TNBC patients as from 26-89-years (Zakaria et al., 2019).

All the 76 TNBC patients in the current study had completed six cycles of TAC chemotherapy regimen. Among these, 25 (32.9%) presented with recurrence. This is almost close to the recurrence rate of 35.0% reported by Pogoda et al., (2013) but higher than the recurrence rate of 17.9% by Dogra et al., (2014) and 16.0% by van Roozendaal et al., (2016) in TNBC patients. In another study by Qiu et al., (2016), the frequency of recurrence or metastasis was higher in TNBC as compared to non-TNBC patients (27.95% vs 13.38% respectively). All these reports are in agreement with the present study which showed a higher recurrence rate in TNBC patients.

TNBC is associated with high histological grade, stage and positive axillary lymph nodes metastasis. In this study, a significant difference in recurrence rate was observed between TNBC patients with axillary lymph nodes metastasis and those with negative axillary lymph nodes metastasis status (P=0.003). This result is comparable with the study conducted by He et al., (2017) that reported high number of positive axillary lymph nodes metastasis associated with poor clinical outcome and low survival rate in TNBC patients. These authors concluded that high numbers of positive axillary lymph nodes metastasis could be a prognostic factor for TNBC patient’s survival rate. In addition, Singh et al., (2020) showed that age ≤ 35, pathological stage, nodal status at diagnosis, perineural invasion and number of positive lymph nodes are associated with recurrence in Indian TNBC patients.

Kaplan-Meier analysis showed that TNBC patients who carried *CYP1B1 4326 GG* genotype were significantly associated with lower DFS as compared to TNBC patients who carried *CYP1B1 4326 CC* or *CG* genotypes (37% vs 71% respectively). Moreover, univariate and multivariate Cox regression analysis demonstrated that carriers of *CYP1B1 4326 GG* genotype had significantly higher risk of relapse with HR: 2.50 and HR: 4.18 even after adjustment. This is in concordance with a few other reports which indicated that *CYP1B1 4326 G *allele was associated with a lower response rate, progressive free survival rate (PFS) and decreased overall survival in breast cancer patients treated with taxanes (Marsh et al., 2007). Next, carriers of two copies of *CYP1B1 4326 G* allele had significantly shorter overall survival (OS) in castration-resistant prostate cancer (CRPC) patients treated with docetaxel-based regimen (Sissung et al., 2008). However, a study by Gehrmann et al., (2008) on breast cancer patients who received treatment with paclitaxel, showed that those with *CYP1B1 4326 GG *genotype had a longer PFS rate as compared to patients with at least one G allele. Rizzo et al., (2010) demonstrated that variant genotype/allele of *CYP1B1 4326 C>G *polymorphism reduced the sensitivity reaction (7 folds) to taxane treatment as compared to wildtype genotype in breast cancer patients. Likewise, studies on other cancers, such as in prostate cancer, Pastina et al., (2010) found a significant association between *CYP1B1 4326 GG *genotype with non-response to the docetaxel (26.1% vs 73.9%, p=0.014) compared to *CYP1B1 4326 CC* and *CG *genotypes. Besides that, carriers of *CYP1B1 4326 GG *genotype was also associated with shorter PFS and overall survival (OS) in non-small-cell lung carcinoma (NSCLC) patients treated with docetaxel as compared to carriers of *CYP1B1 4326 CC* and *CG* genotypes (Vasile et al., 2015).

*CYP1B1 4326 C>G* polymorphism is located at coding sequence of *CYP1B1* mRNA which changes the amino acid from leucine to valine. Studies have demonstrated that *CYP1B1 4326 GG* genotype increased catalytic activity of *CYP1B1* (Landi et al., 2005) and also reduced the sensitivity to chemotherapeutic agents such as interacting agents, alkylator, topoisomerase II inhibitor and antimetabolite (Laroche-Clary et al., 2010). Presence of *CYP1B1 4326 GG* genotype increased the production of 4-OHE2, that has a potential carcinogenic effect by creating DNA adducts, generating reactive oxygen species and finally altering and interfering with drug’s microtubule stabilizing action (Sissung et al., 2008; Takemura et al., 2010; Yager, 2012). This might reduce the* CYP1B1* activity and result in tumour initiation or disease recurrence (Liehr, 2000).

The conflicting results between the present study and other previous studies could be due to difference in the genetic background of the study subjects, type of treatment and drugs used, limited number of study subjects, difference in the type of diseases studied as well as cancer subtypes. Moreover, some studies used a different model for comparison such as dominant model (BB+AB vs AA) or recessive model (BB vs AB+AA) to associate the genotype pattern and patient’s survival. All these factors might have contributed for the inconsistency of results observed in the current study, with various other studies. 

In conclusion, carriers of *CYP1B1 4326 GG* were found to be associated with poor treatment response and increased risk of disease recurrence among Malaysian TNBC patients undergoing TAC chemotherapy. Thus, *CYP1B1 4326 GG* genotype could be considered as a potential biomarker in predicting the risk of disease recurrence in TNBC patients undergoing TAC chemotherapy regimen.

## Author Contribution Statement

Ahmad Aizat Abdul Aziz carried out the experiment, analysed the data and wrote the manuscript. Md Salzihan Md Salleh was involved in pathological confirmation of the cases, Maya Mazuwin Yahya (Breast cancer surgeon) and Andee Dzulkarnaen Zakaria (Breast cancer surgeon) helped in identification and recruitment of TNBC patients and collection of clinical and pathological data. Ravindran Ankathil conceived the idea, designed the study, reviewed manuscript and supervised the project
